# Decomposition of air conditioning electricity consumption considering the dependence between the temperature and electricity consumption

**DOI:** 10.1371/journal.pone.0308542

**Published:** 2024-08-29

**Authors:** Chao Xun, Lin Liu, Chang Wang, Junhong Ni

**Affiliations:** 1 State Grid Fujian Electric Power Co., Ltd., Fuzhou, China; 2 Institute of Economic and Technology, State Grid Fujian Electric Power Co., Ltd., Fuzhou, China; 3 Department of Electronic and Communication Engineering, North China Electric Power University, Baoding, China; RMIT University, AUSTRALIA

## Abstract

With the continuous increase in air conditioning installations, the proportion of air conditioning electricity consumption to total electricity consumption is growing. Research on the decomposition of air conditioning electricity consumption is of great significance for assessing electricity usage, formulating electricity scheduling plans, and ensuring the stable operation of the power grid. Currently, typical day selection strategies for the decomposition of air conditioning electricity consumption often overlook the corresponding relationship between typical daily electricity consumption and temperature. Therefore, this paper proposes an air conditioning electricity consumption decomposition method based on the dependence between temperature and electricity consumption. This method filters typical days based on Copula-based dependence indicators and equiprobable ellipses, determines the baseline electricity consumption curve through model selection voting, and ultimately calculates the air conditioning electricity consumption. The effectiveness of the proposed method is validated by applying it to electricity consumption data in Fuzhou from 2019 to 2022.

## Introduction

Air conditioners are high-energy-consuming devices commonly used in residential homes, office buildings, shopping malls, and other locations. According to data from the National Bureau of Statistics, by the end of 2022, there were 133.9 air conditioning units per hundred households in China, indicating a rising prevalence of air conditioning. It is foreseeable that, with societal development, the electricity consumption of air conditioners will significantly impact the total electricity consumption [1]. This will, in turn, have an increasingly important effect on the economic and stable operation of the power grid and the balance of electricity supply and demand. Therefore, accurately calculating air conditioner electricity consumption can help power companies promptly forecast grid load and electricity sales, enabling timely grid dispatch and ensuring stable grid operation [2]. Given current practical constraints, real-time monitoring and statistical analysis of air conditioner electricity consumption are impractical. Consequently, studying methods for decomposing air conditioner electricity consumption holds significant engineering application value.

Air conditioner electricity consumption is the accumulation of air conditioner load over time; therefore, the methods for decomposing air conditioner load are also applicable to decomposing air conditioner electricity consumption. The practical steps involve decomposing the total electricity consumption in a region based on statistical data from the power grid to calculate air conditioner electricity consumption. Currently, widely used methods for calculating air conditioner load include the basic-load comparing method (BLCM) [3, 4] and the peak-load comparison method (PLCM) [5, 6].

The BLCM calculates the average load curve of working days in spring and autumn to determine the baseline load. At this point, the difference between the daily load curve and the baseline load is the air conditioning load. Reference [2] used the BLCM to separate the air conditioning load curve from the regional load variation curve and employed the Pearson correlation coefficient to examine the relationship between various influencing factors and the air conditioning load, thereby establishing an air conditioning load forecasting model to predict the summer air conditioning load.Reference [7] focused on the relationship between air conditioning load and temperature in Changsha to understand the impact of temperature on electricity load in the region. The study used the BLCM to calculate the cooling load in summer and the heating load in winter and performed a nonlinear regression analysis of the correlation between air conditioning load and daily maximum and minimum temperatures over the past five years. Similarly, references [8] and [9] viewed total power load as comprising baseline load and air conditioning load and made predictions for air conditioning load.Reference [10] applied the BLCM to calculate air conditioning electricity consumption to study the impact of temperature on air conditioning load in the region and used regression analysis to establish a mathematical model for air conditioning load, exploring the relationship between air conditioning load and maximum temperature.

The PLCM identifies the peak load situation in the third quarter’s historical data and compares it with the peak load in months with minimal air conditioning load to determine the maximum cooling load, which is the air conditioning load. Reference [6] studied the practical use of the PLCM and found that the method exhibited a lag effect. To address this, an improved method was proposed. This improved method used the analysis results of the temperature sensitivity of summer air conditioning load to adjust historical electricity load data for temperature variations. The adjusted data were then used to perform curve fitting on the maximum daily load data before applying the PLCM. The improved PLCM can calculate air conditioning load without requiring data for the entire year, thus mitigating the lag effect to some extent.

The advantages of these two methods are their simple calculation processes, but they also have some drawbacks:

Both the PLCM and the BLCM use load data from the spring and autumn seasons as the non-air conditioning load data. This means that for analyzing summer air conditioning load, it is necessary to wait until the end of autumn of the same year, resulting in a significant delay that undermines the timeliness of the guidance;

In practical calculations, both methods fail to adequately account for the natural growth in electricity consumption. Simply averaging the load data from the spring and autumn seasons overlooks the impact of natural growth in electricity consumption. Additionally, the selection of typical months and typical days is subjective and lacks support from objective data. These factors lead to deviations in the final calculation results of air conditioning load.

Reference [6] proposed improvements to address the lag issue, but the strategy for selecting typical days lacks an objective quantitative basis. Reference [11] used the correlation coefficient between temperature and load to screen data for typical days in spring and autumn and fitted the baseline load curve using the screened load data, then decomposed the air conditioning load based on this curve. Although this method considers the impact of temperature on air conditioning load, the correlation coefficient can only describe the linear relationship between temperature and load. In practical engineering, the relationship between temperature and air conditioning electricity consumption is often nonlinear, leading to poor generalization ability for correlation coefficient-based methods.

To address these issues, this paper proposes a method that considers the dependence between temperature and air conditioning electricity consumption. The specific contributions of this paper are as follows:

A strategy for selecting typical days is proposed. The quality of the initial set of typical days is evaluated using a dependence measure based on Copula. Under the assumption of a Gaussian joint distribution, an equal-probability ellipse is constructed using temperature data and the initial set of typical days, and outliers in the typical days are filtered based on the equal-probability ellipse;

Based on the set of typical days filtered for outliers, various regression models are employed to fit the baseline curve of electricity consumption. Through a voting mechanism based on three model selection criteria, the optimal baseline curve is determined, facilitating the calculation of air conditioning electricity consumption;

The method is applied to calculate air conditioning electricity consumption using actual electricity consumption data from Fujian Province. The effectiveness of the proposed method is validated through comparative experiments.

## Typical day selection

Air conditioning electricity consumption mainly comprises the power consumed for cooling in summer and heating in winter. In the spring and autumn seasons, when temperatures are relatively moderate, it is commonly assumed that there is no air conditioning electricity consumption [12]. Therefore, spring and autumn dates are typically selected as typical days in various common air conditioning electricity calculation methods, such as the Basic Load Comparing Method and the Peak Load Comparison Method. However, this selection strategy has notable shortcomings. For instance, in southern regions of China, there may be occurrences of late spring cold snaps or unseasonably cool weather during the spring and autumn seasons. This results in the need for air conditioning to regulate indoor temperatures during certain periods, leading to electricity consumption. Therefore, it is necessary to further refine the strategy for selecting typical days.

**Fig 1:**
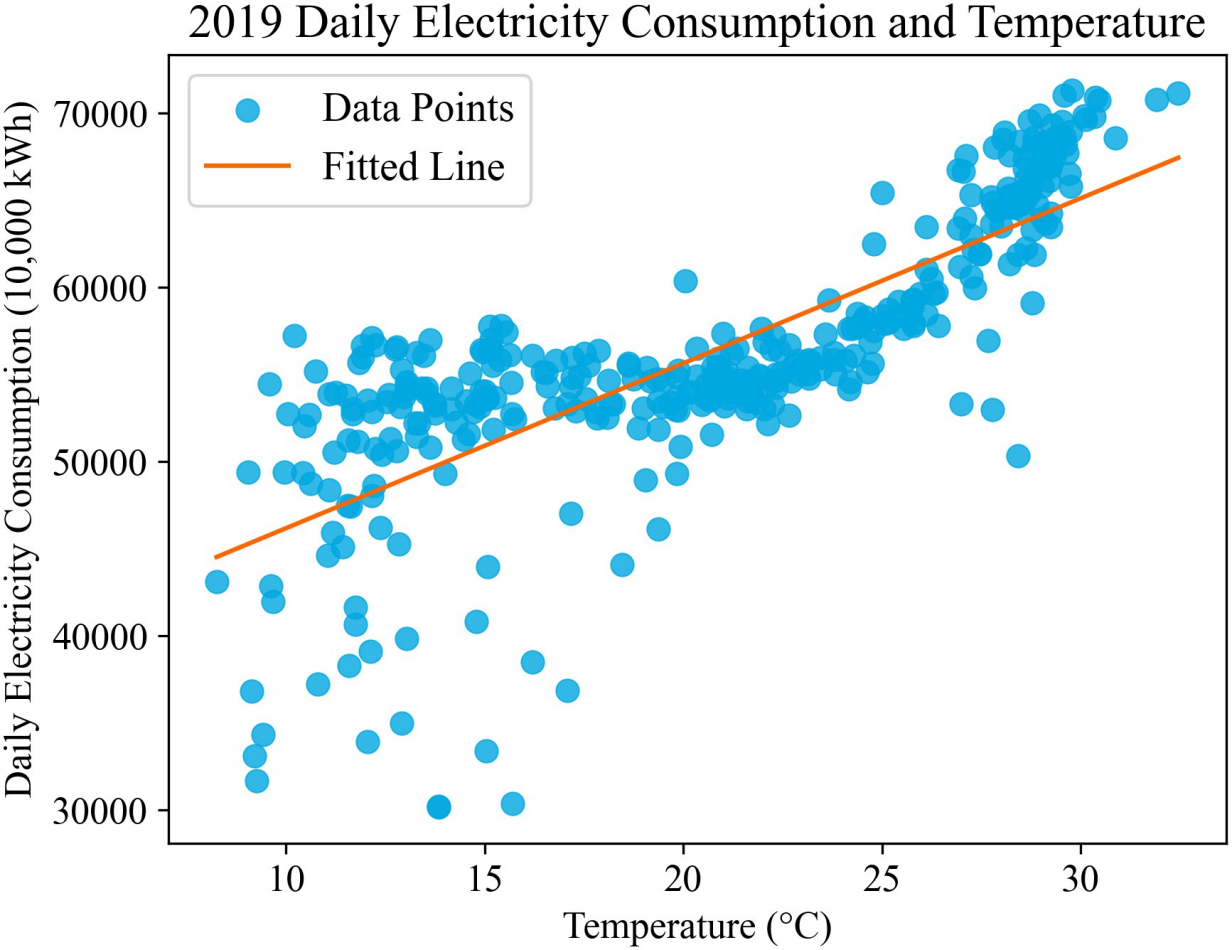
Daily electricity consumption and temperature curve for the year 2019.

Reference [11] evaluates the quality of a set of typical days based on the absolute value of the Pearson correlation coefficient between temperature and the electricity consumption of typical days. This is used to determine whether further filtering of typical days is needed. However, the Pearson correlation coefficient can only describe linear relationships between random variables, while the relationship between temperature and electricity consumption is often nonlinear, as illustrated in Fig 1:

The Fig 1 depicts the distribution of daily electricity consumption data for the year 2019. It can be observed that there is no linear relationship between daily electricity consumption and temperature. In other words, the quantitative relationship between electricity consumption and temperature should not be simplified as linear. Therefore, it is necessary to evaluate the quality of the set of typical days based on indicators that can describe nonlinear relationships between random variables, and subsequently design a method for filtering the set of typical days.

In this paper, the dependency indicator SWσ [13] is used to measure the degree of dependence between daily electricity consumption and its corresponding temperature. The specific calculation formula is as follows:

$$\sigma \left(X_{1},X_{2}\right)=12\int_{{\left[0,1\right]}^{2}}|\text{C}(v)-\;\Pi \;(v)|\text{d}v$$ (1)

Where *X*_1_ and *X*_2_ represent random variables corresponding to typical daily electricity consumption and temperature, respectively, Vector **v** ∈ [0, 1]^2^, C(**v**) represents the copula of *X*_1_ and *X*_2_. Π(**v**) is the product of the distribution functions of *X*_1_ and *X*_2_. Therefore, the symbol *σ* characterizes the distance between the Copula of *X*_1_ and *X*_2_ and the product of their distributions. 0 ≤ *σ* ≤ 1, with larger values of *σ* indicating a higher degree of dependence between *X*_1_ and *X*_2_. The dependency index SWσ, which separates marginal distributions from correlations, is introduced to model the non-linear relationship between random variables. This approach overcomes the limitation of the Pearson correlation coefficient, which only measures linear correlation coefficients.

**Fig 2:**
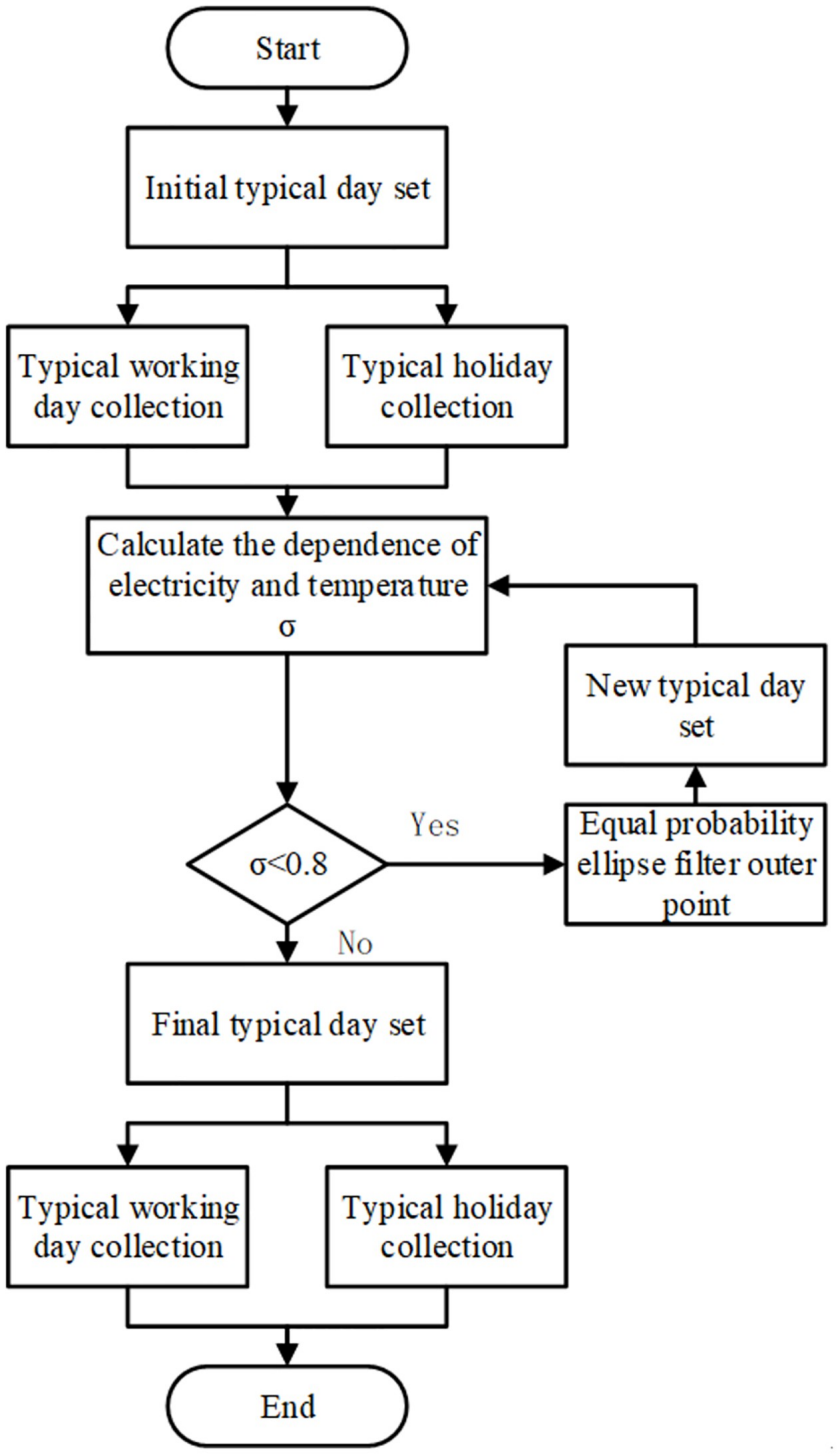
Typical day selection process.

Based on the dependency index SWσ, this paper proposes a typical day selection strategy based on the dependence between temperature and electricity consumption, as outlined in Fig 2:

Identify March as the typical month for spring and November as the typical month for autumn. All dates in March and November are considered typical days, forming the initial set of typical days Ω;

Use the dependency index SWσ to calculate the dependence between temperature and electricity consumption for the initial set of typical days. If the result is below the empirical threshold η, proceed to step 3.

Construct equiprobable ellipses based on the temperature and electricity consumption of the typical days. Filter the typical days based on the equiprobable ellipses.

In Step 2, the computation of the dependency index SWσ involves estimating the copula between temperature and electricity consumption on typical days [14]. In this paper, we employ an empirical formula to estimate the copula function, given by

$$\hat{C}(v_{1},v_{2})=\frac{1}{N}\sum_{i=1}^{N}1({\hat{F}}_{1}(x_{1}^{i})\leq v_{1},{\hat{F}}_{2}(x_{2}^{i})\leq v_{2})$$ (2)

Where,

$${\hat{F}}_{j}(x)=\frac{1}{N}\sum_{i=1}^{N}1(x_{j}^{i}\leq x)$$ (3)

$\left\{x_{j}^{i}|i=1,\ldots ,N\right\}$ represents the observed values of *X*_*j*_, ${\hat{F}}_{j}(x)$ is the estimated distribution function of the random variable *X*_*j*_, *v*_1_ and *v*_2_ represent the two variables for which the Copula needs to be calculated. In this paper, they refer to temperature and typical daily power consumption. The threshold *η* for the dependency index is set to 0.8, indicating that when the dependency index *σ* > 0.8, the dependence between the set of typical days and temperature is considered significant, and there is no need for outlier filtering in the typical day set. Otherwise, if the overall consistency of the set of typical days is poor (i.e., when the dependency index is less than or equal to 0.8), suggesting the presence of outliers, equiprobable ellipses are constructed to filter outlying days in the set.

Under the assumption that the joint distribution of temperature and electricity consumption on typical days follows a Gaussian distribution, the equiprobable ellipse is constructed as follows:

$$\left[X_{1}-{\bar{X}}_{1},X_{2}-{\bar{X}}_{2}\right]M^{-1}{\left[X_{1}-{\bar{X}}_{1},X_{2}-{\bar{X}}_{2}\right]}^{T}=2r^{2}$$ (4)

Here, ${\bar{X}}_{1}$ and ${\bar{X}}_{2}$ represent the means of *X*_1_ and *X*_2_, respectively, and **M**^−1^ is the inverse matrix of the cross-correlation matrix

$$M=\frac{1}{N}\sum_{i=1}^{N}{\left[x_{1}^{i}-{\bar{X}}_{1},x_{2}^{i}-{\bar{X}}_{2}\right]}^{T}\left[x_{1}^{i}-{\bar{X}}_{1},x_{2}^{i}-{\bar{X}}_{2}\right]$$ (5)

Given a confidence level *γ* for outliers in typical days, the parameter *r* is obtained by solving the following equation:

$$\int_{-r}^{r}\frac{1}{2\pi }e^{-\frac{1}{2}z^{2}}dz=\sqrt[{2}]{1-\gamma }$$ (6)

Eq (6) can be solved using the standard normal distribution lookup table. The probability of typical days falling outside the equiprobable ellipse is given by

$$P\left({\left[\begin{array}{l} X_{1}-{\bar{X}}_{1}\\ X_{2}-{\bar{X}}_{2} \end{array}\right]}^{T}M^{-1}\left[\begin{array}{l} X_{1}-{\bar{X}}_{1}\\ X_{2}-{\bar{X}}_{2} \end{array}\right]>2r^{2}\right)<\gamma$$ (7)

**Fig 3:**
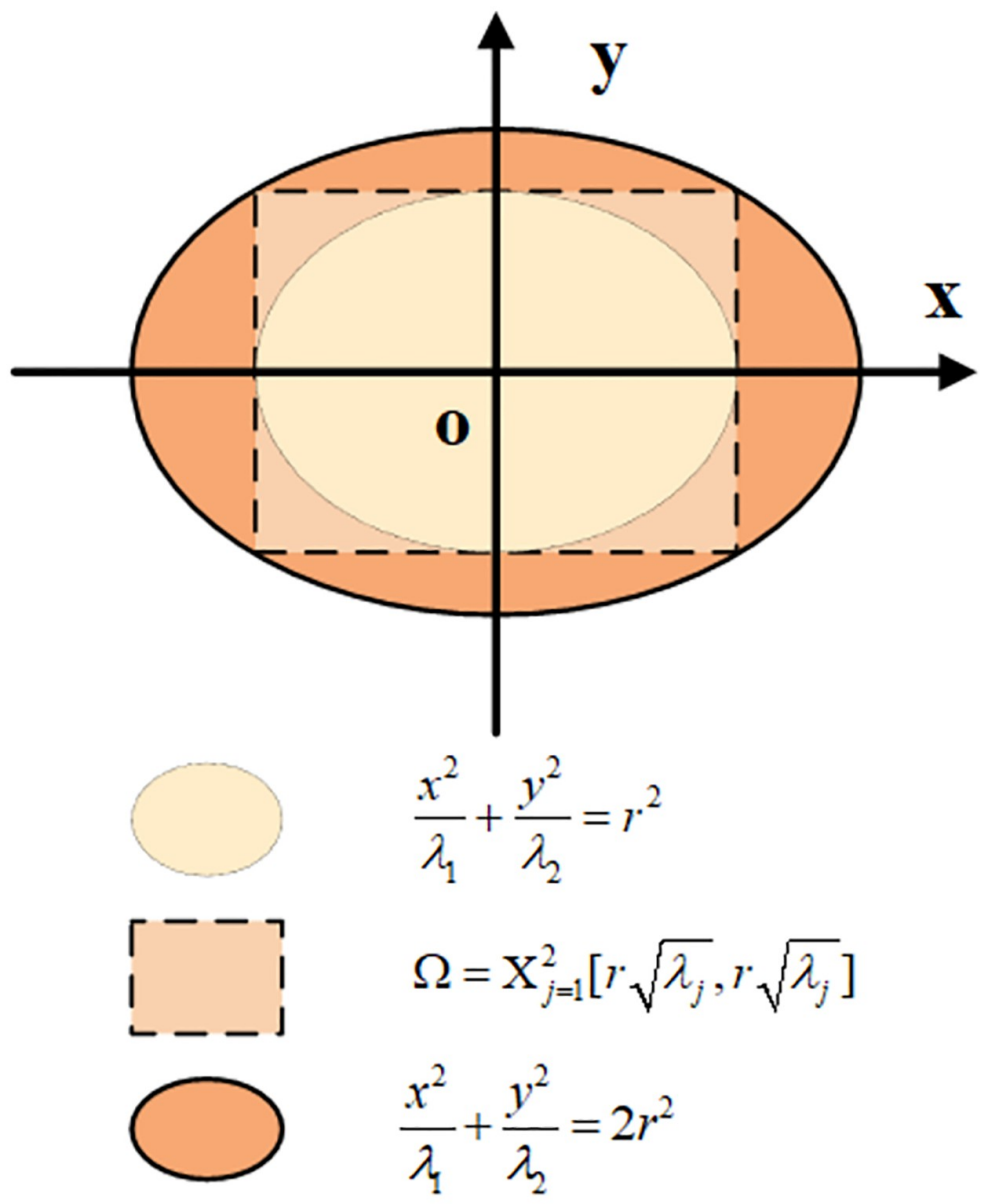
Equiprobable ellipse.

In this study, the confidence level *γ* is set at 5%. The equiprobable ellipse is illustrated in Fig 3:

In Fig 3, *λ*_1_ and *λ*_2_ represent the eigenvalues of the cross-correlation matrix **M**, the dashed rectangle is the circumscribing rectangle of the tan-colored ellipse, and the circumscribing ellipse of the circumscribing rectangle (ocher-colored ellipse) is the equiprobable ellipse.

## Baseline electricity consumption fitting

On the basis of the identified set of typical days in the previous section, a regression analysis approach [15, 16] is employed to fit the baseline electricity consumption curve. This involves determining the baseline electricity consumption curve by fitting a regression curve to the set of typical days against time labels.

Various regression models have been reported in the literature, including polynomial models, exponential models, logarithmic models, etc. In reference [11], a method for model selection is proposed, calculating the coefficient of determination (R^2^) for each model and choosing the model with the highest R^2^ as the optimal regression model to fit the baseline electricity consumption curve. While this approach is straightforward, its limitation lies in the simplicity of the model selection criterion, often failing to identify the actual optimal regression model. Therefore, in this paper, a combination of multiple model selection criteria is used to vote for the optimal regression model.

The model selection criteria employed in this study include the Akaike Information Criterion (AIC) [17], Bayesian Information Criterion (BIC) [18, 19], and Deviance Information Criterion (DIC) [20].

Four reference regression models are employed, including linear model, quadratic model, exponential model, and logarithmic model:

$$P_{1}=j_{1}t+k_{1}$$ (8)

$$P_{2}=j_{2}t^{2}+k_{2}t+l_{2}$$ (9)

$$P_{3}=j_{3}e^{k_{3}t}$$ (10)

$$P_{4}=j_{4}t+k_{4}\;\text{ln}\;t$$ (11)

Where *j*_1_, *j*_2_, *j*_3_, *j*_4_, *k*_1_, *k*_2_, *k*_3_, *k*_4_ and *l*_2_ are the parameters to be estimated, which can be estimated using the least squares method [21].

**Fig 4:**
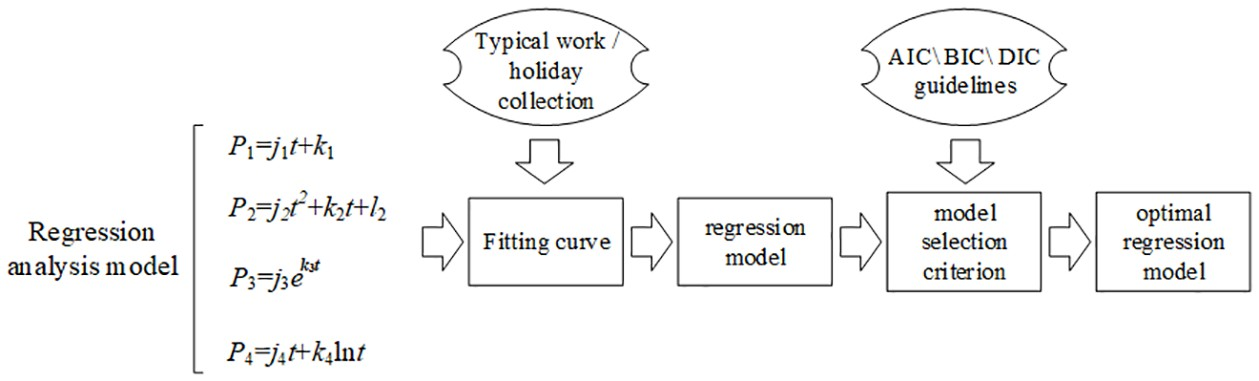
Flowchart for baseline electricity consumption curve selection.

The voting process for selecting the optimal regression model is conducted following the procedure illustrated in Fig 4.

First, calculate the scores under the AIC, BIC, and DIC criteria for the four regression models, denoted as $C_{i}^{AIC}$, $C_{i}^{BIC}$, and $C_{i}^{DIC}$, *i* = 1, 2, 3, 4; Second, normalize the scores of the four regression models under the same model selection criterion. Finally, compute the sum of scores for each regression model under all model selection criteria. The ultimate score for a regression model is then given by:

$$C_{j}=\frac{\frac{1}{3}C_{j}^{AIC}}{\sum_{i=1}^{4}C_{i}^{AIC}}+\frac{\frac{1}{3}C_{j}^{BIC}}{\sum_{i=1}^{4}C_{i}^{BIC}}+\frac{\frac{1}{3}C_{j}^{DIC}}{\sum_{i=1}^{4}C_{i}^{DIC}}$$ (12)

where *C*_*j*_ ∈ [0, 1] is referred to as the weighted model selection index. Smaller scores under the mentioned model selection criteria and closer values of the weighted model selection index to 0 indicate better performance of the corresponding regression model. Therefore, the regression model with the minimum score in Eq (12) is considered the optimal model.

Furthermore, due to the significant difference in electricity consumption between weekdays and weekends, this paper subdivides the set of typical days into typical weekdays and typical weekends, and selects the optimal model to fit the corresponding baseline electricity consumption curve for each subset. Here, "weekends" refers to days when people rest, including both non-working days and holidays.

## Air conditioning electricity decomposition

**Fig 5:**
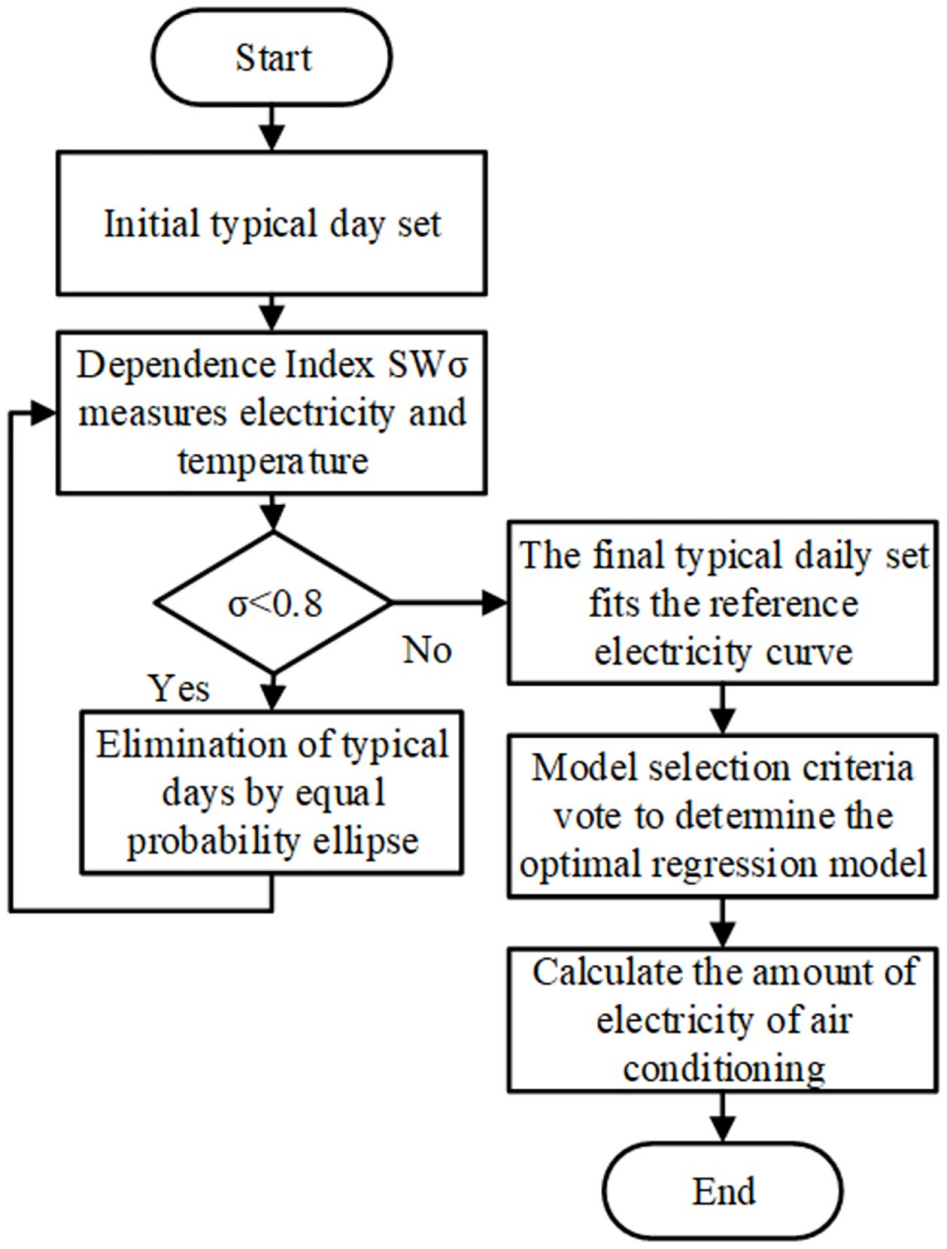
Flowchart for air conditioning electricity consumption decomposition.

Based on the baseline electricity consumption curve obtained from the fitting calculations in the previous section, the overall process for decomposing daily air conditioning electricity consumption is illustrated in Fig 5, as follows:

Select all dates in spring and autumn as the initial set of typical days. Filter the typical days using the dependency index between temperature and electricity consumption to obtain subsets for typical weekdays and typical weekends.

Employ the model selection criteria for voting to determine the optimal regression model and fit the baseline electricity consumption curve.

Calculate the difference between total electricity consumption and baseline electricity consumption as the final air conditioning electricity consumption

$$P_{\text{AIR}}=P_{\text{L}}-P_{\text{B}}$$ (13)

Where *P*_AIR_, *P*_L_, and *P*_B_ represent air conditioning electricity consumption, total electricity consumption, and baseline electricity consumption, respectively.

## Calculation example

### Example introduction

**Fig 6:**
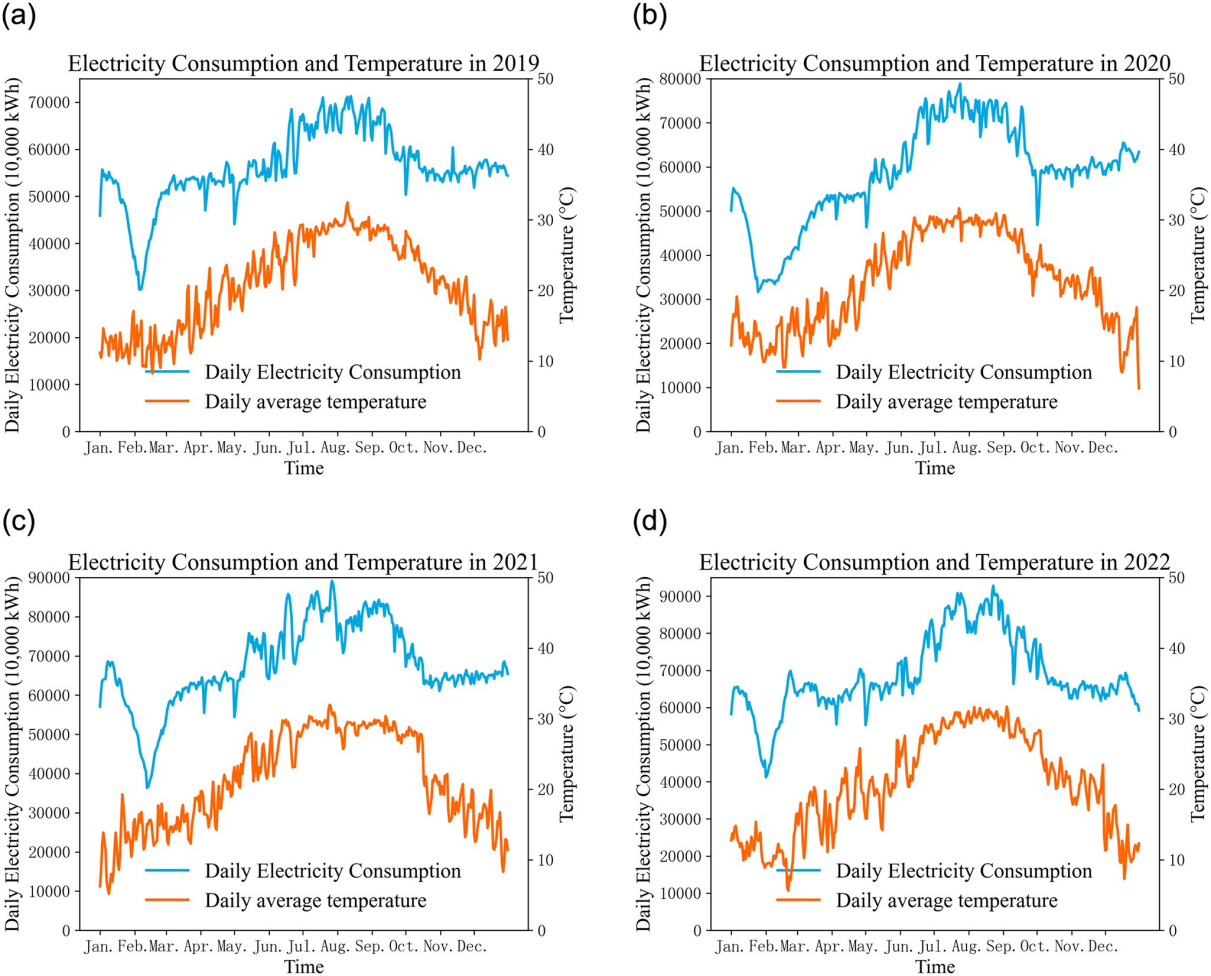
Curve chart of electricity consumption and temperature in Fuzhou City.

This section involves data collected from Fuzhou, Fujian Province, China. Fuzhou has a typical subtropical maritime monsoon climate, characterized by warm and humid weather. The summer season is hot and is the period with the highest air conditioning electricity consumption throughout the year. While winters are not extremely cold, prolonged periods of cool weather may still result in some heating-related air conditioning electricity consumption. The daily electricity consumption curves for Fuzhou from 2019 to 2022 are shown in Fig 6. It can be observed that during each summer, daily electricity consumption and daily average temperature are at their peaks. From October to December each year, despite a gradual decrease in temperature, daily electricity consumption generally remains at a consistent level. In winter, starting from January, daily electricity consumption undergoes a phase of initial decline followed by an increase.

### Typical day selection

This section designates the days in March and November, which almost have no air conditioning electricity consumption throughout the year, as the initial set of typical days. Based on the typical day selection strategy outlined in the previously mentioned section on Typical Day Selection, which is dependent on the relationship between temperature and electricity consumption, the initial set of typical days is further divided into weekdays and weekends, and typical day selection is performed separately.

Table 1 presents the results of typical day selection for the year 2019. To better illustrate the effectiveness of the proposed typical day selection strategy, Table 1 specifically lists the excluded specific typical days, along with their corresponding electricity and temperature values. The table also provides the dependency index between electricity consumption and temperature for the typical day set before and after the selection. It can be observed that the excluded typical days either have exceptionally high electricity values (possibly due to significant data collection errors) or temperatures below the comfort range (in this paper, the temperature range where air conditioning cooling or heating is unnecessary is [20°C, 25°C]). After the selection, the dependency index between the typical day set and the corresponding temperature significantly increases.

**Table 1:** 2019 typical day selection results.

Typical daily set	Typical day to be excluded	Electric quantity (10000kWh)	Temperature (°C)	Dependency index before and after screening	
				before	after
weekdays	2019.03.07	53955.67	11.25	0.65	0.80
	2019.03.08	54443.49	9.58		
	2019.11.01	53394.33	21.83		
	2019.11.04	53848.38	21.71		
weekends	2019.03.03	50422.93	12.42	0.68	0.82
	2019.11.02	53807.33	21.63		
	2019.11.03	53019.35	22.04		
	2019.11.17	53004.67	21.58		

The screening statistics for the typical day set comprising all typical days from 2019 to 2022 are shown in Table 2. It is observed that in 2020, the dependency between daily electricity consumption and daily average temperature is relatively high, and no exclusion is necessary to meet the threshold. This indicates that extreme weather events in March and November 2020 occurred less frequently compared to the other three years. In 2021, a total of 3 days were excluded, and the anomalous dates were mainly concentrated on weekdays in November. This is attributed to a sudden temperature drop on several days without a significant change in daily electricity consumption, adversely affecting the dependency index. In 2019 and 2022, 8 days and 7 days were excluded, respectively, with the excluded dates relatively concentrated. This is primarily due to meteorological factors causing sudden temperature changes on certain days, resulting in differences in temperature compared to adjacent dates. However, the electricity consumption did not exhibit a significant short-term variation, affecting the numerical value of the dependency index. For example, in 2022, most of the exclusions occurred in March when the region experienced widespread low-temperature rain and snowfall in spring. People in the local area rarely use air conditioning in spring, leading to a deterioration in the consistency between daily electricity consumption and temperature.

**Table 2:** 2019–2022 typical day selection statistics.

Year	Typical daily set	Culling days	Dependency index
2019	weekdays	4	0.80
	weekends	4	0.82
2020	weekdays	0	0.92
	weekends	0	0.80
2021	weekdays	3	0.81
	weekends	0	0.95
2022	weekdays	6	0.84
	weekends	1	0.80

**Fig 7:**
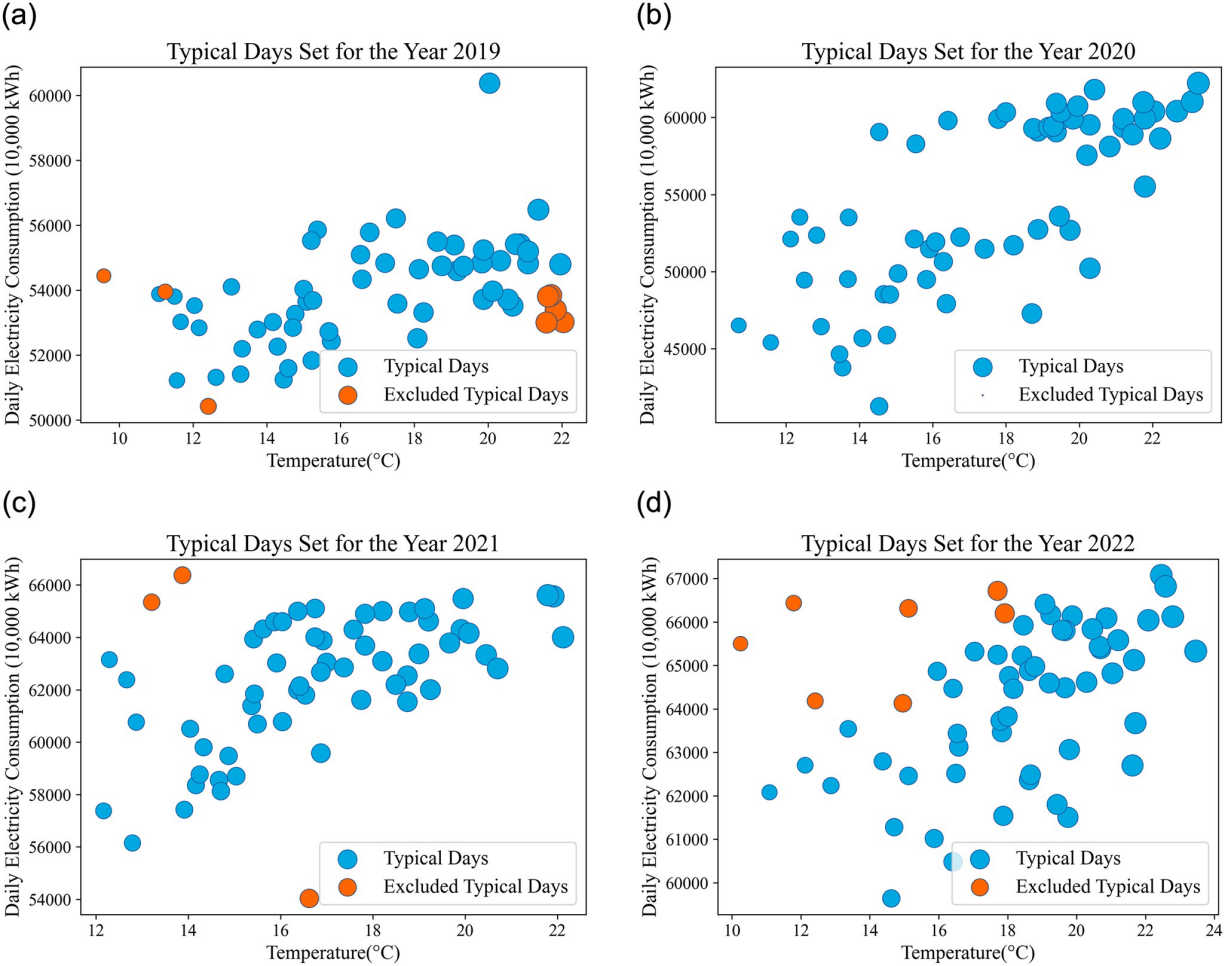
Bubble chart of typical daily selections from 2019 to 2022.

For ease of observation, the visual representation of the typical day set and the excluded anomalous typical days is shown in Fig 7. In the graph, the x-axis represents the temperature of the typical days, and the y-axis represents the electricity consumption of the typical days. It is evident that the physically meaningful excluded typical days are outliers.

### Fitting of baseline electricity consumption curve

**Fig 8:**
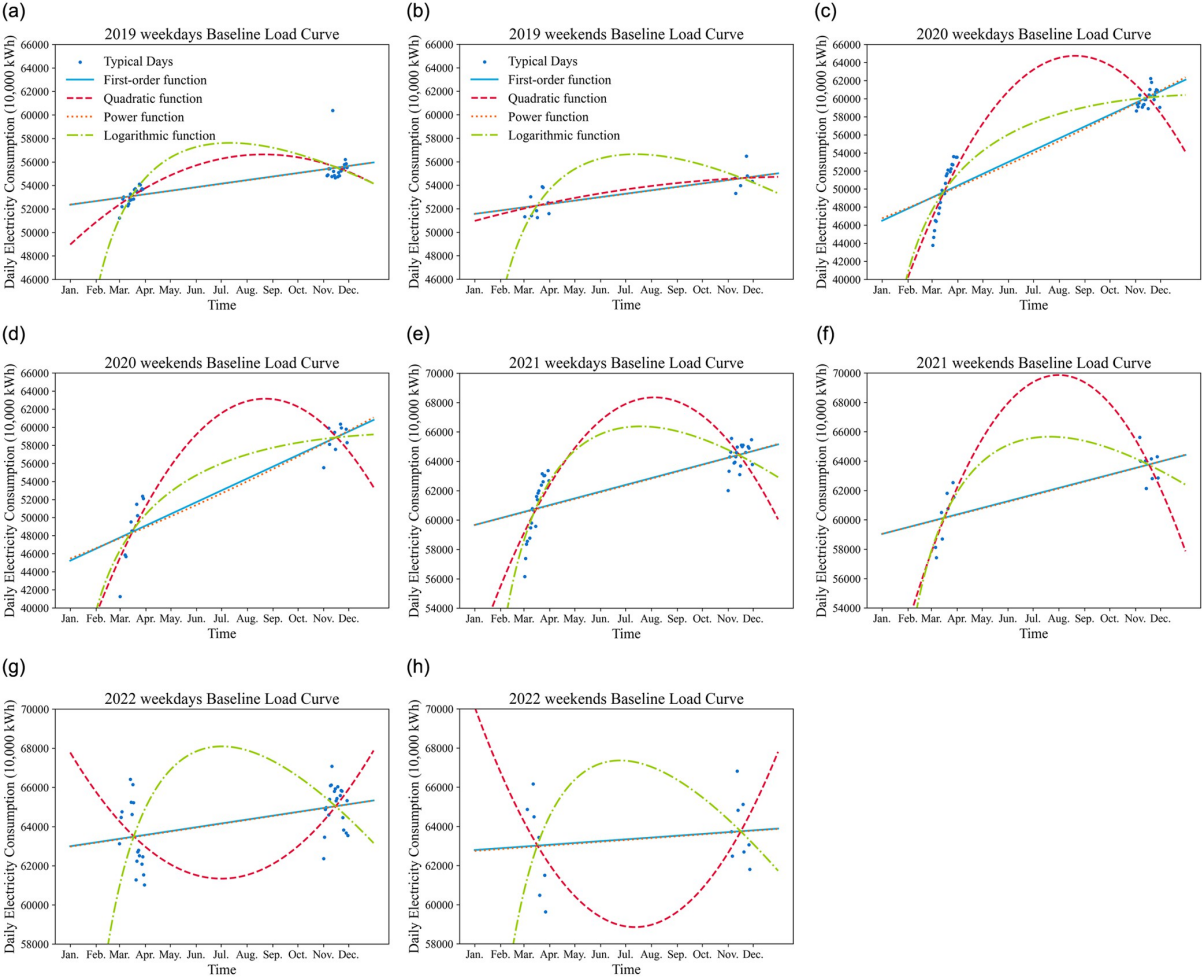
Fitted curve of benchmark electricity consumption from 2019 to 2022.

The fitted baseline electricity consumption curves for the typical weekdays and typical weekends, based on the filtered sets for the years 2019 to 2022, are illustrated in Fig 8.

For comparison purposes, the fitting curves for all four regression models are plotted in the graph. Table 3 provides the selection results of regression models during the fitting process for the baseline electricity consumption in 2019–2022. In 2019, the power function model was chosen as the final optimal regression model for fitting the baseline curve on weekdays, while the logarithmic function model was selected as the final optimal regression model for fitting the baseline curve on weekends. In both 2020 and 2021, the power function model was chosen as the optimal regression model for fitting both weekday and weekend baseline curves. In 2022, the logarithmic function model was selected as the optimal regression model for both weekday and weekend baseline curves.

**Table 3:** Results of regression model selection of benchmark curve from 2019 to 2022.

	Selection criteria	AIC	BIC	DIC	Our criterion
weekdays (2019)	First-order function	633.1	634.7	1262.2	0.25051
	Quadratic function	629.8	631.4	1255.6	0.24921
	Power function	633.1	634.8	1262.3	0.25053
	Logarithmic function	631.2	632.8	1258.4	0.24976
weekends (2019)	First-order function	250.5	251.2	497.1	0.24894
	Quadratic function	250.5	251.2	497.0	0.24890
	Power function	250.5	251.3	497.1	0.24895
	Logarithmic function	254.8	255.5	505.6	0.25320
weekdays (2020)	First-order function	776.5	778.3	1549.0	0.25381
	Quadratic function	753.1	754.8	1502.2	0.24615
	Power function	777.6	779.3	1551.1	0.25416
	Logarithmic function	752.2	754.0	1500.5	0.24588
weekends (2020)	First-order function	332.9	333.8	661.8	0.25251
	Quadratic function	326.7	327.5	649.3	0.24777
	Power function	333.2	334.1	662.5	0.25278
	Logarithmic function	325.5	326.4	647.1	0.24693
weekdays (2021)	First-order function	734.6	736.3	1465.1	0.25422
	Quadratic function	719.4	721.1	1434.7	0.24896
	Power function	734.7	736.5	1465.4	0.25428
	Logarithmic function	700.8	702.6	1397.6	0.24254
weekends (2021)	First-order function	279.0	279.8	554.0	0.25542
	Quadratic function	267.5	268.3	531.0	0.24489
	Power function	279.0	279.8	554.1	0.25548
	Logarithmic function	266.7	267.5	529.5	0.24420
weekdays (2022)	First-order function	677.3	679.0	1350.7	0.24825
	Quadratic function	673.4	675.1	1342.9	0.24682
	Power function	677.3	679.0	1350.7	0.24825
	Logarithmic function	700.3	702.0	1396.7	0.25668
weekends (2022)	First-order function	271.6	272.3	539.1	0.24896
	Quadratic function	270.2	270.9	536.5	0.24774
	Power function	271.6	272.3	539.1	0.24897
	Logarithmic function	277.4	278.1	550.8	0.25433

### Calculation of air conditioning electricity consumption

**Fig 9:**
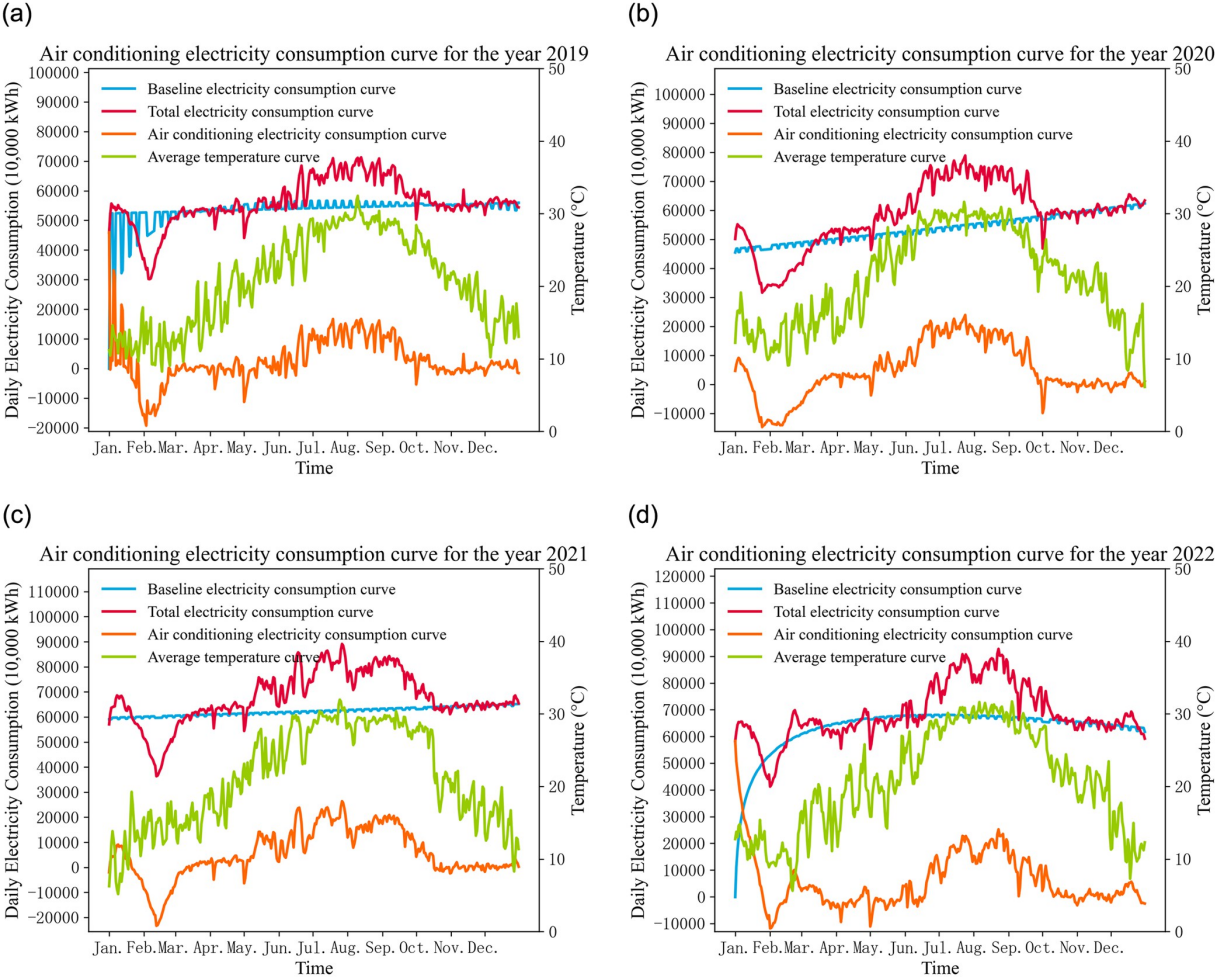
Curve chart of air conditioning electricity consumption from 2019 to 2022.

Subtracting the baseline electricity consumption from the total electricity consumption yields the final air conditioning electricity consumption. Fig 9 illustrates the air conditioning electricity consumption curves decomposed using the method proposed in this paper for the years 2019 to 2022.

Observing the air conditioning electricity consumption curve and the temperature curve in Fig 9, it is evident that their trends are roughly similar. This similarity is primarily due to the fact that the impact of temperature on air conditioning electricity consumption is most pronounced, especially in summer, where higher temperatures result in greater air conditioning electricity consumption.

**Fig 10:**
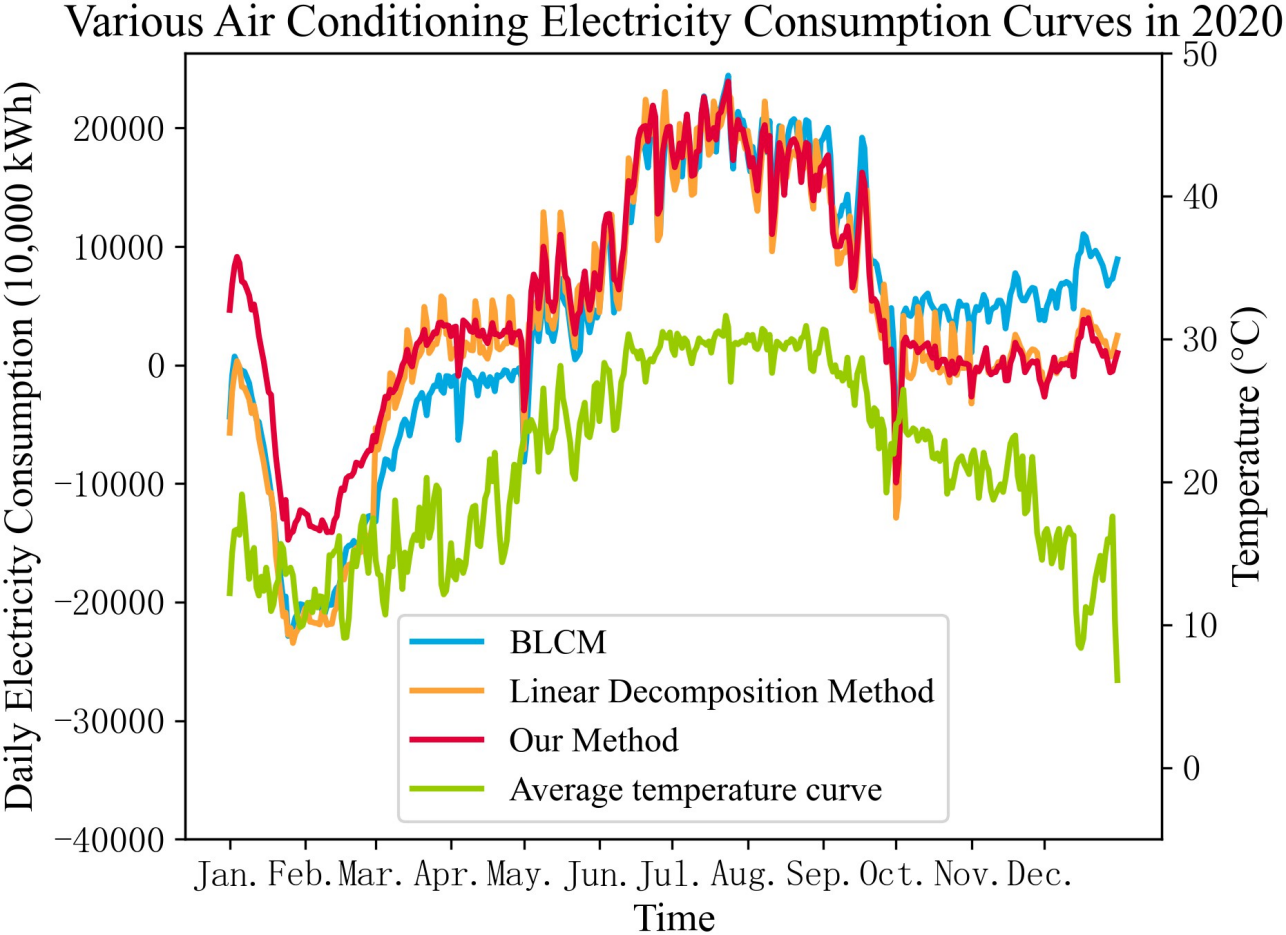
Air conditioning electricity consumption calculated by different methods.

To validate the effectiveness of the proposed method considering the temperature-electricity dependency, the baseline load comparison method and the linear decomposition method were employed to calculate the air conditioning electricity consumption for Fuzhou from 2019 to 2022. The results were then compared with the method proposed in this paper. Fig 10 displays the calculated air conditioning electricity consumption results for the year 2020 using the three different methods. As observed, the three curves exhibit the same trend. However, compared to the method proposed in this paper, the two comparison methods yield relatively larger fluctuations in air conditioning electricity consumption. This is because both the baseline load comparison method and the linear decomposition method choose all typical days to fit the baseline curve, leading to a higher occurrence of negative values in the calculated air conditioning electricity consumption, particularly in the January to April period.

The occurrences of negative values in air conditioning electricity consumption calculated by the three methods are presented in Table 4.

**Table 4:** Daily proportion of negative electricity of air conditioners in 2020.

Calculation method of electricity quantity of air conditioner	Proportion of negative electricity of air conditioner
Reference load comparison method	34.15%
Linear decomposition method	31.15%
Our method	26.78%

The proposed method benefits from the filtering of typical days and the optimal regression model voting selection, resulting in the lowest proportion of negative air conditioning values. Additionally, the dependency indices for the three methods are relatively close, as shown in Table 5.

**Table 5:** Dependence index of three air conditioning decomposition methods.

Calculation method of electricity quantity of air conditioner	Dependency index
Reference load comparison method	0.96
Linear decomposition method	0.99
Our method	0.96

Although the linear decomposition method exhibits higher dependency, its calculation results include too many negative values in air conditioning electricity consumption, which deviates from real-life scenarios. In contrast, the proposed method not only yields fewer negative values in air conditioning electricity consumption but also demonstrates superior dependency indices, validating the effectiveness and practicality of this method. Thanks to the advantages of the decomposition method presented in this paper, effective decomposition of air conditioning electricity consumption can be achieved. This is significantly beneficial for understanding the air conditioning electricity consumption in a given region. By comprehending the variations in air conditioning electricity consumption, power grid companies can better ensure the supply of electricity in the region and maintain the stable operation of the power grid system.

## Conclusions

This paper investigates the unsupervised decomposition problem of air conditioning electricity consumption and proposes a decomposition method based on typical day selection and regression curve voting. This method selects typical days using an equiprobable ellipse, and designs a voting method to choose the optimal regression model for fitting the baseline electricity consumption curve, thereby achieving the decomposition of air conditioning electricity consumption. Experimental results on real electricity consumption data validate the effectiveness and practicality of the proposed decomposition method. By separating the air conditioning electricity consumption from the overall electricity data, power grid companies can understand the air conditioning electricity usage in a region. This enables them to promptly and accurately adjust the power supply composition and distribution during special periods, such as extreme summer heat or sudden winter cold. Understanding air conditioning electricity consumption is also an important component of understanding regional electricity usage, which is of great significance in real-life applications.

## Supporting information

S1 File (ZIP)
